# Department of Gynecology

**Published:** 1894-04

**Authors:** 

**Affiliations:** F. R. C. S., Ed., Professor of Anatomy University of Texas; late Physician for Diseases of Women, Edinburgh Providence Dispensary; Galveston


### DEPARTMENT OE GYNECOLOGY.

EDITED BY WM. KEILLER, F. R. C. S., ED.,
Professor of Anatomy University of Texas; late Physician for Diseases of Women, Edinburgh Providence Dispensary.

THE DIRECT EXAMINATION OF THE FEMALE BLADDER.—Howard A. Kelly, *Am. Jour. Obstet. and Dis. Wom.*, January, '94.

Dr. Kelly describes a simple method by which he can, by dilating the urethra, examine directly the whole inner surface of the female bladder, including the orifices of the ureters, and by which the ureters can be catheterised under direct inspection.

The patient, being placed in the lithotomy position, the bladder is emptied, the urethra dilated by graduated dialators, a tubular speculum introduced, residual urine sucked up, and the interior of the bladder examined directly, the light being thrown in from a good window, or electric or argand light, by a head mirror, such as is used in throat examination. The whole of the interior of the bladder can thus be examined, and catheters introduced into either or both ureters. No anæsthetic is necessary. Thus obscure bladder cases may be clearly diagnosed; each ureter may be examined as to the character of the secretion from the corresponding kidney, or bougies may be introduced into the ureters prior to hysterectomy. Medicaments may be applied through the speculum directly to the diseased area in the bladder wall, or forceps, snares, etc., used under direct vision. The method is simple, and the inconvenience to the patient slight. It is likely to be a valuable method of diagnosis and treatment.

---

DISPUTED POINTS IN THE SURGERY AND PATHOLOGY OF PELVIC DISEASE.—Jos. Price, *Am. Jour. Obstet. and Dis. Wom.*

Beruntz & Goupil's work proved long ago the fatal effects of temporizing in pelvic suppuration; puerperal peritonitis is often due to the rupture of a pus containing tube during labor, yet in face of these facts some men advise rest and temporizing in pyosalpynx, and urge as a reason that thus the tube may again become functional! And if it do regain its function, such tubes are one of the causes of tubal pregnancy! Such methods as fixing the uterus by a pin thrust through the abdominal wall, or aspirating a pyosalpynx through vagina or abdomen, are as dangerous and as bad surgery, as it would be to tie the carotid by boldly thrusting the needle through skin and fasciæ. Puncture and drainage of pelvic cellular tissue abscesses is uncertain, they are often multilocular; vaginal opening of such abscesses is frequently unsuccessful, for they can not be thoroughly evacuated.

The causes of suppurative disease of tubes and ovaries are gonorrhæa in the male, dirty widwifery, abortion with retained placental debris. "Catching cold" is mythical, unless coming on the top of an antecedent inflammation.

Tubes and ovaries are disorganized and rendered functionally useless; and bowel, omentum and bladder are involved in adhesive inflammation, pain, and sometimes obstruction resulting. If, then, the pathology be definite, so should the surgery be. Hopelessly diseased parts, and parts interfering with the function of more important organs, should be removed. As to the after-effects of the removal of tubes and ovaries, the benefits are real and lasting where there has been marked cause for their removal, doubtful only where they have been removed for lesions barely justifying interference.

Where insanity follows their removal, it would most likely have come on at the climacteric in any case.

The abdominal surgeon must have a thorough surgical training. There is a feeling that any one may open the abdomen, though it requires a specialist to remove a cataract.

---

The other day, while taking lunch at a restaurant, a young physician came in and sat down opposite to me at the table. He made my acquaintance, and said he had graduated three months previously. He also said he had located an office, and presented me with his new card, proudly. To my astonishment, he deliberately announced himself as on a determined hunt to find a laparotomy case, and to do it himself. I asked him whether he had ever done laparotomy? He replied, "no." "Did you ever do a post mortem?" He said, "no, but he had dissected his 'quarter of the belly' when in college." I talked with him a half hour, and tried to show him that he was unjust to himself and patient. But he replied that his teachers had taught him that laparotomy was one of the easiest of surgical operations. * * * An unfortunate new school of laparotomists is rising. Young men who have done no general practice, who know neither anatomy nor pathology; men who go to doing a gynecological practice and abdominal surgery, right from college. They never did general surgery; they just become specialists from the day they leave a medical college. Such specialists are limited in their views, biased in practice, and dangerous in abdominal surgery.—*Dr. F. Byron Robinson* (*at Chicago Gynec. Socy.*)

---

### PEDIATRICS.

THE ALIMENTATION OF INFANTS.—Budin (Paris) *International Clinics*, Vol. IV., 1894.

In La Charité maternity hospital two charts are kept, one of the mother's temperature, the other of the daily weight of the child. Daily weighing is the best means of testing the child's progress.

The child loses 100–300 grammes (3½ to 8½ ounces) during the first two days, then the weight increases, so that it is back to the original weight by the seventh day and 100 grammes heavier than at birth on the tenth day. The rate of increase is about 9 drachms (35 grammes) per day.

If the mother has not enough milk, the child may continue to lose weight after the second day. Such children do not cry, nurse little, sleep all the time and are said to be "doing well." They pass very little urine and rapidly get emaciated. They need careful watching and the mother's milk must be supplemented with artificial feeding.

The amount of milk which the child gets from the mother may be estimated by weighing the child before and after each nursing. She ought to secrete twelve to sixteen ounces daily (in first month). Wet nurses being expensive and difficult to procure, ass's milk was tried in the hospital, but ultimately cow's milk, sterilized, was found to give the best result. It is given where necessary during the first two or three days to supplement the mother's milk, and gradually withdrawn or continued, according to the amount of her secretion; or given alone where the mother does not nurse. As a test of the efficiency of the nourishment, a child doing well should increase twenty grammes (5 drachms) per day. (Average result in 191 cases.) Results seem to show that *properly sterilized* cow's milk is as readily digested and assimilated and gives as good results as mother's milk, but everything depends on its proper preparation.

Of eighty-nine children, nursed by mothers, six had diarrhœa; of ninety-two on mother's milk supplemented by sterilized milk, seven had diarrhœa; of eleven on sterilized milk alone, none had diarrhœa. All the cases of diarrhœa were mild. Budin does not dilute the cow's milk with water but gives it *pure* from the very first.

Sterilization so modifies the milk that it coagulates in small curds like mother's milk instead of large masses like unboiled cow's milk. The children take less of the pure milk but gain weight more rapidly than those on diluted milk.

Woman's milk contains 877 parts water to 123 solids; cow's milk, 865 parts water to 135 solids; then as they approach so closely, why dilute so largely as is the custom?

Budin places the milk to be sterized in small bottles, each containing enough milk for one nursing [ordinarily three or four ounce bottles do excellently.—Ed.]; he covers them with a special rubber cap which permits the exit of steam, but, as soon as the bottle is removed from the boiling water, is sucked in by the vacuum so as to tightly seal the bottle. [It is sufficient to place the open bottle in the boiler, and to cork them with ordinary corks which have been boiled, as soon as the lid is removed and while they are still surrounded by the steam of the sterilizer.—ED.] The bottles are placed in a metal frame to hold six to nine (the number of nursings per diem) and this is put in a suitable pot of cold or warm water, which being covered is then allowed to boil briskly for forty minutes. If the special rubber caps are not used, the bottles are corked firmly while in the boiling water, and then removed. Enough milk is prepared for one day's nursing only; but milk so prepared will keep for a week, even in warm weather. The cork is removed, the bottle warmed, a teat slipped on, and it is ready for use. They and the corks should be thoroughly cleansed and boiled before being used again.

[Any tin-smith can make the sterilizer for fifty cents. A tin can deep enough to hold nine ordinary four ounce bottles, with an inch to spare, a round perforated tin base with four corners turned down to serve as feet and raise it half an inch above the bottom of the boiler, a frame of wire fitted to this with a central handle to support the bottles, (which any handy man can fit on himself) and the apparatus is complete.—ED.]

